# Impact of camel milk lactoferrin peptides against breast cancer cells: *in silico* and *in vitro* study

**DOI:** 10.3389/fphar.2024.1425504

**Published:** 2024-11-19

**Authors:** Othman Baothman, Ehab M. M. Ali, Hassan Alguridi, Salman Hosawi, Emadeldin Hassan E. Konozy, Isam M. Abu Zeid, Abrar Ahmad, Hisham N. Altayb

**Affiliations:** ^1^ Department of Biochemistry, Faculty of Science, King Abdulaziz University, Jeddah, Saudi Arabia; ^2^ Center of Artificial Intelligence in Precision Medicines, King Abdulaziz University, Jeddah, Saudi Arabia; ^3^ Division of Biochemistry, Chemistry Department, Faculty of Science Tanta University, Tanta, Egypt; ^4^ Laboratory of Proteomics and Glycobiology, Biotechnology Department, Africa City of Technology, Khartoum, Sudan; ^5^ Biomedical and Clinical Research Centre (BCRC), College of Health and Allied Sciences (CoHAS), University of Cape Coast, Cape Coast, Ghana; ^6^ Department of Biological Sciences, Faculty of Science, King Abdulaziz University, Jeddah, Saudi Arabia

**Keywords:** HER2 protein, camel milk, MCF-7 breast cancer cells, HDFa normal cells, *in silico*

## Abstract

**Background and Aims:**

Breast cancer remains a significant global health concern, necessitating the exploration of novel therapeutic strategies. Despite advancements in cancer therapeutics, effective treatments with minimal side effects remain elusive. Natural sources, such as camel milk, harbor bioactive compounds such as lactoferrin peptides, which hold promise as anticancer agents. This study investigated the potential of camel milk-derived lactoferrin peptides against breast cancer cells through a combined *in silico* and *in vitro* approach. By integrating computational modeling with experimental assays, we aimed to elucidate the anticancer mechanisms of these peptides and provide insights for their optimization as anticancer therapeutics.

**Methods:**

*In silico* analysis involving pepetid design, and validation, then molecular docking and molecular dynamics (MD) simulations was used to explore peptide-protein interactions and stability. Peptides were synthesized and tested for anticancer activity using MTT assays on MCF-7 cells, with HDFa normal cells used as controls.

**Results:**

Results of this study showed that camel milk-derived lactoferrin peptides, particularly PEP66, exhibited strong anticancer activity against MCF-7 breast cancer cells, with the lowest IC50 value (52.82 μg/mL) compared to other peptides. *In silico* molecular docking and dynamics simulations revealed that PEP66 formed stable interactions with key residues in the HER2 catalytic site, indicating its potential as an effective anticancer agent. The selectivity index (SI) of PEP66 (3.19) also suggested lower toxicity to normal cells compared to cancer cells, reinforcing its therapeutic potential. Hydrogen bonding analysis highlighted key residues involved in stabilizing peptide-protein complexes, while molecular dynamics simulations demonstrated the stability of these interactions over time. Notably, PEP66 exhibited the highest stability and formed significant interactions with essential residues in the HER2 catalytic site, suggesting its potential as an effective anticancer agent.

**Conclusion:**

Camel milk-derived lactoferrin peptides show promise as anticancer agents against breast cancer cells. The multidisciplinary approach employed in this study provides valuable insights into the mechanisms underlying their activity, paving the way for rational design strategies to enhance their efficacy. Further experimental validation is warranted to validate the anticancer potential of these peptides and advance their development as novel therapeutic agents for breast cancer treatment.

## Background

Cancer remains one of the most pressing health challenges worldwide, with millions of lives affected by its devastating consequences annually. According to GLOBOCAN 2022 data estimates, lung cancer was the most frequently diagnosed cancer in 2022, responsible for almost 2.5 million new cases, or one in eight cancers worldwide (12.4% of all cancers globally), followed by cancers of the female breast (11.6%), colorectum (9.6%), prostate (7.3%), and stomach (4.9%) (Bray et al., 2024). Despite significant advancements in cancer treatment, the quest for novel therapeutic agents with enhanced efficacy and reduced side effects continues. In recent years, there has been a burgeoning interest in exploring natural sources as potential reservoirs of bioactive compounds possessing anticancer properties. Protein and peptide medications provide notable benefits in cancer treatment owing to their potency, specificity, multifunctionality, and minimal toxicity. Over the past few decades, there have been remarkable advancements in protein delivery mechanisms, ensuring the protection of these drugs within the body and their effective transportation to tumor locations and cells (Liu et al., 2019). These natural sources offer a promising avenue for the discovery and development of new cancer therapeutics (Asma et al., 2022; Konozy and Osman, 2022; Baothman et al., 2024).

Traditionally acknowledged for its potential health benefits, camel milk has been considered a potential solution for various human ailments, including those associated with conditions such as diabetes (Malik et al., 2012), heart disease (Kaskous, 2016), and even cancer. Research suggests that camel milk exhibits anticancer properties, as evidenced by several studies conducted both in laboratory settings and with live subjects (Krishnankutty et al., 2018; Alkhulaifi et al., 2024). The peptides within camel milk, known for their therapeutic potential, have attracted considerable attention among its diverse natural resources. Lactoferrin (LF), a significant bioactive compound abundant in mammalian milk, has become a primary focus of biomedical research, generating substantial interest (Swelum et al., 2021). LF, a multifunctional iron-binding glycoprotein abundant in mammalian milk, has gained significant attention due to its various biological functions, including antimicrobial, immunomodulatory, and anticancer effects (Superti, 2020; Rascón-Cruz et al., 2021a).

Peptides derived from lactoferrin represent a particularly intriguing area of research in cancer therapy (Superti, 2020; El-Fakharany et al., 2022). These peptides, which are typically short sequences of amino acids, exhibit bioactive properties and have potential as anticancer agents (Khan et al., 2021). Camel milk, a traditional beverage consumed in several regions globally, contains lactoferrin peptides with unique sequences and structures, which may contribute to their enhanced bioactivity compared to peptides from other sources (Superti, 2020; Khan et al., 2021).

HER2 is a protein involved in controlling cell growth and division. MCF-7 cells belong to the Luminal A subtype of breast cancer cells, characterized by the presence of estrogen receptor (ER) and progesterone receptor (PR). When the estrogen receptor is stably overexpressed in MCF-7 breast cancer cells, it leads to increased levels of HER2 mRNA and protein, even in the absence of estradiol. Researchers often use the MCF-7 cell line to investigate the influence of HER2 expression and regulation on cellular behavior, response to treatments, and various aspects of breast cancer biology and therapy (Lattrich et al., 2008).

*In silico* approaches play a crucial role in drug design process (Wadood et al., 2013). Notably, the use of computational techniques, including molecular docking and molecular dynamics (MD) simulations has been increasingly reported. They provide a cost-effective and time-efficient means to screen multiple peptide candidates and predict their binding affinities with target proteins (Saikia and Bordoloi, 2019). Various previous studies have demonstrated the effectiveness of combining *in silico* and *in vitro* approaches for the discovery of potent anticancer peptides from different sources. Taghipour and his colleagues identified anticancer peptides from camel milk protein hydrolysates (Taghipour et al., 2023), Fatemi and his colleague identified anticancer peptides derived from human lactoferrin (Fatemi et al., 2018).

The use of lactoferrin peptides extracted from camel milk is an attractive strategy for enhancing their anticancer efficacy. Chemical modifications can improve peptide stability, bioavailability, and target specificity, thereby overcoming limitations associated with natural peptides (Al Musaimi et al., 2022). Additionally, chemical modifications can modulate the interaction of peptides with cancer cells, leading to enhanced cytotoxicity and the induction of apoptosis (El-Fakharany et al., 2022). Given the potential of lactoferrin peptides derived from camel milk as anticancer agents, there is a compelling rationale to explore their efficacy against breast cancer cells. This research aimed to investigate the anticancer potential of these peptides through a comprehensive approach involving both computational modeling (*in silico*) and laboratory experimentation (*in vitro*). By elucidating the mechanisms underlying the anticancer activity of camel milk-derived lactoferrin peptides, this study seeks to pave the way for their optimization as effective anticancer therapeutics. Through a multidisciplinary approach integrating computational predictions with experimental validation, this research endeavors to contribute to the development of novel strategies for breast cancer treatment.

## Materials and methods

### *In silico* analysis

#### Molecular docking

Molecular docking was conducted to study the possible interactions between the six peptides and the HER2 protein. HER2 was selected due to its high expression in MCF-7 breast cancer cell lines (Kumar et al., 1996). Peptide 3D structures were built in AlphaFold via Structure Prediction of ChimeraX (Meng et al., 2023). The 3D structure of HER2 (PDB ID: 7PCD) was obtained from the PDB database and prepared by removing crystallized ligand water molecules. The peptide docking server (HPEPDOCK 2.0) (Zhou et al., 2018) was used for docking the peptide to the active site of the protein, and the active pocket that contains essential residues was determined according to Wilding et al. (2022). (Wilding et al., 2022). Docking complexes were analyzed and visualized by the Protein-Ligand Interaction Profiler (Adasme et al., 2021). The co-crystalized ligand was used as a control for *in silico* analysis.

#### Molecular dynamics (MD) simulation

MD simulation was used to study the stability of the complex of peptides and the HER2 protein. The Desmond module (Bowers et al., 2006) was employed for these studies. The complexes were prepared for simulation by being placed in a TIP4P water model inside a 10 Å orthorhombic box. The OPLS3e force field was used for system minimization over 2000 iterations. Simulations were carried out under the NPT ensemble at a constant temperature of 300 K (K) and pressure of 1.0133 bar (1.0133 bar). MD simulation was carried out for 50 ns, then data snapshots were captured at 100 ps intervals throughout the MD simulation.

#### Peptide synthesis and *in vitro* anticancer activity

In this study, we designed CM peptides using different computational tools, as described in our previous study (Alguridi et al., 2023). Six peptides (PEP20, PEP24, PEP63, PEP66, PEP67, and PEP74) (Table 1) were predicted to have anticancer activity using the AntiCP V. 2.0 tool (https://webs.iiitd.edu.in/raghava/anticp/multi_pep.php) (Agrawal et al., 2021). These peptides were synthesized commercially by GenScript Biotech Corporation (GenScript, Piscataway, NJ) and were dissolved in ultrapure water at different concentrations ranging from 10 mM to 250 mM to study their anticancer activity.

**TABLE 1:** Peptide sequences and properties.

Peptide ID	Peptide sequence	SVM score	Prediction	Hydrophobicity	Hydropathicity	Hydrophilicity	Charge	Mol wt
PEP67	LFFPALLSLGALGLCLAASK	0.67	Anticp	0.23	1.7	−0.85	1	2005.8
PEP74	QLNQLQGLKSCHTGLGRSAGW	0.77	Anticp	−0.15	−0.52	−0.27	2.5	2,254.9
PEP24	PWNHIKRYF	0.53	Anticp	−0.25	−1.29	−0.48	2.5	1,260.6
PEP63	VAHLEQVLLR	1.08	Anticp	−0.05	0.69	−0.32	0.5	1,177.6
PEP20	CGSIVPRREWRAL	0.87	Anticp	−0.28	−0.3	0.18	2	1,543
PEP66	CSTSPLLEACAFLMR	0.94	Anticp	−0.01	0.85	−0.4	0	1,642.2

The Tissue Culture Unit, Department of Biochemistry, Faculty of Science, King Abdulaziz University, donated MCF-7 (breast cancer) and HDFa (adult human dermal fibroblast). DMEM media high glucose (Cat No 11965092), fetal bovine serum heat inactivated (Cat No A3840301), penicillin-streptomycin (10,000 U/mL) (penicillin-streptomycin (10,000 U/mL, trypsin (0.25%) (Cat No 25050030) were purchased from Gibco (ThermoFisher Scientific, United States). Dulbecco′s Phosphate Buffered Saline (DPBS) (Cat NO. D8537), Thiazolyl Blue Tetrazolium Bromide (MTT) powder (Cat No M5655) were purchased from Sigma-Alderich company (M5655). Dimethyl sulfoxide (DMSO) (Cat No 50-255-891) was purchased from Fisher Scientific Co.

DMEM medium supplemented with 10% fetal bovine serum (FBS) and 1% penicillin-streptomycin (10,000 U/mL) (complete media) was prepared to culture MCF-7 and HDFa cells in T25 flasks at 37°C in a CO_2_ incubator. Once the cells had adhered and grown to 70%–90% confluence within 24–48 h, they were transferred to new flasks. 2 mL of 0.25% trypsin was added to detach the cells, which were then incubated for 5 min at 37°C. The trypsin activity was stopped by adding 2 mL of complete media. The detached cells were collected into a tube and centrifuged at 1,500 rpm for 5 min. After removing the supernatant, 3 mL of media was added to resuspend the cell pellet. 20 μL of suspended cells were mixed with 20 µL of 0.4% trypan blue for staining and cell counting. 100 μL of suspended cells in complete media, containing approximately 10^4^ cells, were added to each well of a 96-well plate and incubated for 24 h to allow cell attachment. Peptides (PEP20, PEP24, PEP63, PEP66, PEP67, and PEP74) at various concentrations (200, 100, 50, 25, 12.5, 6.25 μg/mL) were then added to the media in each well. Each concentration was repeated three times. After a 48-h incubation period at 37°C, the peptide-treated medium was replaced with 100 µL of 0.5 mg/mL MTT and incubated for 4 h at 37°C in the dark. Following removal of the MTT and addition of 100 µL of DMSO, the absorbance of each well in the 96-well plate was measured at 595 nm using an ELISA reader (Bio-RAD microplate reader, Japan) (Ali et al., 2018; Salim et al., 2022).

The selectivity index (SI) was calculated to determine the toxicity of the peptides to normal cells. SI = IC_50_ cancer cells/IC_50_ normal cells. An SI greater than 2 indicates that the peptide is the least toxic to normal cells (El-Bendary et al., 2024).

#### Statistical analysis

The percentage viability was calculated by multiplying the absorbance of treated cells by 100 and dividing the absorbance of untreated cells. The viability of untreated cells is considered 100%. The each concentration of each peptide and cisplatin was repeated triplicate. The unite of cell viability and IC_50_ are percentage and μg/mL. The viability and IC_50_ of the treated cells are expressed as the mean ± standard deviation (SD). GraphPad Prism software (version 9.0, San Diego, CA, United States) was used to calculate the IC_50_ values of the peptides, mitotane, and their combination.

## Results and discussion

### *In silico* anticancer analysis

The peptides listed in Table 1 are predicted to have anticancer properties (Anticp) based on various parameters, including the support vector machine (SVM) score, hydrophobicity, hydropathicity, hydrophilicity, charge, and molecular weight (Mol wt). The SVM scores indicate the likelihood of anticancer activity, with higher scores suggesting stronger potential (Teerasak et al., 2016). Hydrophobicity and hydropathicity values reflect how peptides interact with water and biological membranes, which are crucial for their function in the body. Hydrophilicity and charge affect a peptide’s solubility and interaction with cellular targets (Badani et al., 2014). The molecular weight provides insight into the size of the peptide, which affects its biological availability and transport. PEP67 shows moderate anticancer potential with a balanced profile of hydrophobicity and hydropathicity, indicating that it may easily interact with cell membranes. PEP74 and PEP20 have greater charges, suggesting that they can interact strongly with negatively charged cellular components (Sitaram and Nagaraj, 1999). Among the listed peptides, PEP63 had the highest SVM score, indicating that it has the strongest anticancer potential (Boopathi et al., 2019). PEP66 shows a good balance in its properties, making it a promising candidate for further investigation. The variance in the properties of these peptides underscores the importance of a multifaceted approach for predicting peptide functionality and emphasizes the potential of using SVM and other physicochemical properties for identifying promising anticancer peptides.

### Molecular docking

**FIGURE 1:**
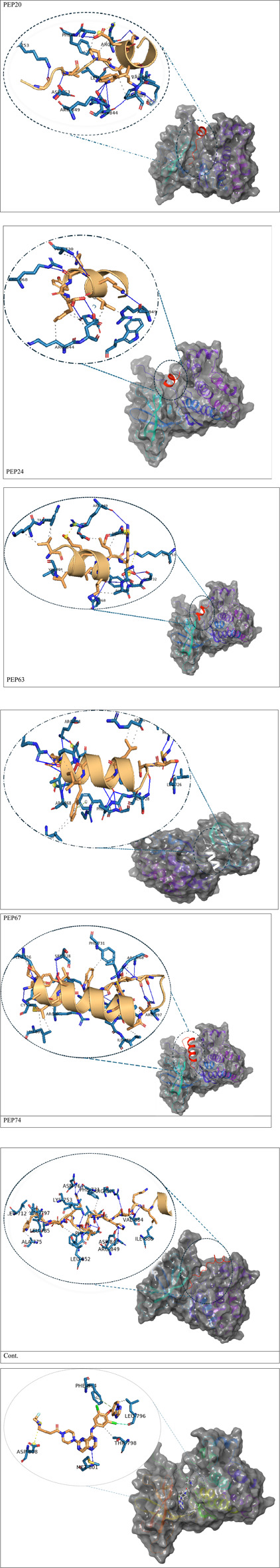
Detailed 3D and 2D interactions of peptides and the control (centers of small and large circles) and HER2 residues (black); blue lines indicate hydrogen bonds, yellow dashed lines indicate π-cation interactions and gray dashed lines indicate hydrophobic bonds. Enlarged figures were generated with the Protein‒Ligand Interaction Profiler (Adasme et al., 2021).

Table 2 and Figure 1 provide data on the hydrophobic bonds between the peptides (PEP24, PEP63, PEP67, and PEP66) and HER2. Each peptide is associated with specific amino acid (AA) residues, distances, ligand atoms, and protein atoms. PEP24 interacts with HER2 at positions 730A, 844A, 845A, 885A, and 888A, corresponding to residues ALA, ARG, ASP, PRO, and TRP, respectively. The distances ranged from 3.52 to 3.96 Å PEP63 binds to HER2 at positions 730A, 845A, 863A, 884A, 887A, and 888A. The residues included ALA, ASP, VAL, LYS, and TRP. The distances vary from 2.46 to 3.88 Å PEP67 interacts with HER2 at multiple positions, including at positions 726A, 731A, 805A, 807A, 845A, 849A, 852A, 862A, 863A, 868A, and 884A, corresponding to residues comprising LEU, PHE, CYS, LEU, ASP, ARG, LEU, THR, ASP, ARG, and VAL, respectively. Their distances range from 2.31 to 3.92 Å.

**TABLE 2:** Hydrophobic bonds generated from the interaction of HER2 with peptides (PEP24, PEP63, PEP67, and PEP76).

Peptide	Index	Residue	AA	Distance	Ligand atom	Protein atom
PEP20	1	730A	ALA	3.17	71	567
	2	731A	PHE	3.68	70	582
	3	731A	PHE	3.27	71	580
	4	868A	ARG	3.99	70	2,748
	5	884A	VAL	3.57	85	2,854
	6	885A	PRO	3.86	148	2,870
PEP24	1	730A	ALA	3.83	118	528
	2	844A	ARG	3.76	166	2,327
	3	845A	ASP	3.52	161	2,351
	4	885A	PRO	3.96	165	2,832
	5	885A	PRO	3.58	77	2,831
	6	888A	TRP	3.63	79	2,894
PEP63	1	730A	ALA	3.03	102	525
	2	845A	ASP	3.76	138	2,349
	3	863A	ASP	3.26	136	2,638
	4	884A	VAL	2.65	50	2,809
	5	887A	LYS	3.42	7	2,863
	6	888A	TRP	2.46	6	2,890
	7	888A	TRP	3.32	5	2,892
PEP67	1	726A	LEU	2.66	28	663
	2	731A	PHE	3.23	344	720
	3	805A	CYS	3.03	52	1872
	4	807A	LEU	3.86	53	1902
	5	807A	LEU	2.96	54	1905
	6	845A	ASP	3.6	336	2,530
	7	849A	ARG	3.03	51	2,581
	8	852A	LEU	2.31	7	2,638
	9	862A	THR	3.51	9	2,807
	10	863A	ASP	3.88	72	2,819
	11	868A	ARG	3.8	297	2,888
	12	884A	VAL	3.42	164	2,993
	13	884A	VAL	2.52	221	2,995
	14	885A	PRO	3.35	165	3,011
	15	886A	ILE	3.92	211	3,027
PEP66	1	849A	ARG	3.23	89	2,449
	2	849A	ARG	4	87	2,448
	3	866A	LEU	3.53	207	2,725
	4	868A	ARG	2.33	154	2,755
	5	870A	LEU	3.76	157	2,798
	6	870A	LEU	3.85	155	2,799
	7	884A	VAL	3.62	171	2,861
PEP74	1	712A	LEU	3.88	301	353
	2	712A	LEU	3.27	296	355
	3	731A	PHE	3.23	198	678
	4	753A	LYS	3.71	245	1,022
	5	767A	ILE	3.62	244	1,254
	6	772A	TYR	3.39	301	1,332
	7	775A	ALA	3.12	304	1,381
	8	785A	LEU	3.54	296	1,521
	9	785A	LEU	2.42	286	1,518
	10	852A	LEU	3.54	220	2,594
	11	862A	THR	3.56	220	2,763
	12	863A	ASP	3.19	218	2,775
	13	864A	PHE	3.64	279	2,790
	14	866A	LEU	3.56	198	2,815
	15	886A	ILE	3.65	120	2,983
Cont	1	796A	LEU	3.67	2,206	662
	2	798A	THR	3.80	2,223	677

Hydrogen bonds play essential roles in stabilizing the structures of biological molecules such as proteins and nucleic acids, as well as in various intermolecular interactions (Tan et al., 2021). As shown in Table 3 and Figure 1, different hydrogen bond interactions were generated between HER2 and the peptides PEP24, PEP63, PEP67, and PEP66. Each peptide interacts with specific residues of HER2, represented by amino acids and their corresponding numbers. For example, PEP24 forms hydrogen bonds with ALA730, ASP845, ARG849, ASP863, and ARG868. PEP63 interacts with GLY729, ALA730, PHE731, GLY732, LYS753, ARG849, ASP863, LEU866, and ARG868. PEP67 forms hydrogen bonds with LEU726, SER728, CYS805, HIS843, ARG844, ASP845, ASP863, LEU866, and ARG868. Finally, PEP66 interacted via hydrogen bonds with LEU726 with significant bond lengths and angles, indicating a specific spatial orientation. SER728A and GLY729A show tight bonding characteristics, with short distances and large angles, suggesting strong interactions. The repeated appearance of ARG897A with different bonding characteristics highlights its potential importance in interaction dynamics. Five residues (Arg849, Leu866, Arg868, Leu870, and Val884) are associated with stable hydrogen interactions with PEP66.

**TABLE 3:** Hydrogen bonds generated from the interaction of HER2 with peptides (PEP24, PEP63, PEP67, and PEP66).

Peptide	Index	Residue	AA	Distance H-A	Distance D-A	Donor angle	Donor atom	Acceptor atom
PEP20	1	730A	ALA	1.93	2.74	133.88	563 [Nam]	136 [O.co2]
	2	753A	LYS	1.77	2.7	150.03	928 [N3+]	16 [O3]
	3	844A	ARG	3.28	3.85	117.19	88 [Ng+]	2,365 [O2]
	4	845A	ASP	1.67	2.7	166.23	153 [Npl]	2,393 [O.co2]
	5	849A	ARG	3.3	3.81	112.34	2,444 [Ng+]	153 [Npl]
	6	849A	ARG	3.43	3.95	111.86	2,447 [Ng+]	153 [Npl]
	7	863A	ASP	1.89	2.51	121.39	2,681 [O3]	31 [Nam]
	8	863A	ASP	1.51	2.51	157.56	31 [Nam]	2,681 [O3]
	9	866A	LEU	3.05	3.81	132.46	80 [Nam]	2,717 [O2]
	10	868A	ARG	2.51	2.89	100.96	112 [Ng+]	2,746 [O2]
	11	884A	VAL	2.64	3.66	174.3	2,849 [N3]	146 [O2]
	12	893A	SER	3.44	3.9	110.08	89 [Ng+]	3,009 [O2]
	13	897A	ARG	3.06	3.6	114.3	3,086 [Ng+]	87 [Ng+]
PEP24	1	730A	ALA	3.12	3.96	141.33	524 [Nam]	94 [O2]
	2	845A	ASP	2.28	3.24	174.8	168 [O2]	2,354 [O.co2]
	3	849A	ARG	2.11	3.09	163.97	99 [N3]	2,401 [O2]
	4	863A	ASP	2.46	3.06	120.36	169 [O2]	2,639 [O2]
	5	868A	ARG	2.88	3.85	162.28	2,714 [Ng+]	137 [Nam]
	6	868A	ARG	2.18	2.99	136.31	2,711 [Ng+]	116 [O2]
PEP63	1	729A	GLY	2.72	3.32	118.26	514 [Nam]	162 [O2]
	2	730A	ALA	3.31	4.07	134.06	521 [Nam]	100 [O2]
	3	731A	PHE	1.55	2.5	155.91	531 [Nam]	161 [O2]
	4	732A	GLY	1.15	2.11	154.87	551 [Nam]	162 [O2]
	5	732A	GLY	1.9	2.55	122.78	162 [O2]	554 [O2]
	6	753A	LYS	2.65	3.66	177.7	886 [N3+]	151 [Nam]
	7	849A	ARG	3.4	3.77	103.71	160 [Ng+]	2,399 [O2]
	8	863A	ASP	3.08	3.59	116.14	2,640 [O3]	135 [O2]
	9	866A	LEU	2.75	3.55	137.23	2,673 [Nam]	116 [O2]
	10	868A	ARG	2.71	3.59	144.36	2,709 [Ng+]	100 [O2]
PEP67	1	726A	LEU	3.46	3.92	109.78	37 [Nam]	662 [O3]
	2	728A	SER	2.98	3.94	159.51	685 [Nam]	90 [O3]
	3	805A	CYS	2.32	3.06	129.1	1868 [Nam]	1 [N3]
	4	843A	HIS	3.63	3.96	101.89	318 [Ng+]	2,488 [O2]
	5	844A	ARG	2.38	2.83	107.11	319 [Ng+]	2,505 [O3]
	6	844A	ARG	1.01	1.73	141.06	2,505 [O3]	318 [Ng+]
	7	845A	ASP	2.21	2.9	128.48	346 [O2]	2,532 [O2]
	8	845A	ASP	2.64	3.58	154.71	2,533 [O-]	312 [O2]
	9	863A	ASP	2.55	3.46	158.69	347 [O2]	2,818 [O2]
	10	866A	LEU	2.77	3.63	136.69	2,857 [O3]	309 [Nam]
	11	868A	ARG	2.49	3.31	138.26	2,890 [Ng+]	296 [O2]
	12	868A	ARG	2.79	3.74	158.01	2,883 [Nam]	293 [Nam]
	13	868A	ARG	3.11	3.48	102.86	293 [Nam]	2,890 [Ng+]
	14	884A	VAL	3.2	4.05	141.7	2,989 [N3]	177 [Nam]
	15	897A	ARG	3.52	3.99	111.19	3,228 [Ng+]	287 [O3]
	16	897A	ARG	3.12	4.06	155.47	3,229 [Ng+]	285 [O2]
PEP66	1	726A	LEU	2.39	2.98	119.24	18 [O3]	529 [O2]
	2	728A	SER	1.87	2.55	120.94	552 [Nam]	27 [O2]
	3	729A	GLY	3.64	4.05	106.6	563 [N3]	108 [O.co2]
	4	729A	GLY	3.29	4.05	139.56	108 [O.co2]	563 [N3]
	5	730A	ALA	3.08	4.05	160.64	570 [Nam]	104 [O2]
	6	731A	PHE	2.25	3.1	140.19	580 [Nam]	108 [O.co2]
	7	732A	GLY	2.18	3.05	142.1	600 [Nam]	108 [O.co2]
	8	766A	GLU	3.01	3.98	161.75	212 [Ng+]	1,147 [O2]
	9	801A	MET	2.25	3.26	176.7	1,676 [Nam]	6 [S3]
	10	801A	MET	2.62	3.45	140.15	1 [N3]	1,679 [O2]
	11	844A	ARG	2.41	3.13	127.59	211 [Ng+]	2,379 [Ng+]
	12	866A	LEU	2.43	3.29	142.68	203 [Nam]	2,724 [O2]
	13	866A	LEU	1.52	2.39	139.47	2,721 [Nam]	140 [O2]
	14	867A	ALA	3.11	3.48	102.46	2,740 [Nam]	206 [O2]
	15	868A	ARG	2.2	2.85	121.22	2,750 [Nam]	150 [O2]
	16	897A	ARG	3.24	3.83	118.86	3,095 [Ng+]	170 [O2]
	17	897A	ARG	1.74	2.74	164.01	3,096 [Ng+]	170 [O2]
PEP74	1	731A	PHE	2.51	3.07	113.69	248 [Ng+]	672 [O2]
	2	753A	LYS	2.47	3.48	171.36	1,024 [N3+]	233 [N3]
	3	764A	ASN	1.83	2.79	153.26	249 [Ng+]	1,203 [O2]
	4	797A	VAL	2.32	3.23	157.87	269 [O3]	1702 [O2]
	5	845A	ASP	1.59	2.64	171.76	183 [Npl]	2,489 [O.co2]
	6	849A	ARG	2.83	3.2	101.66	2,540 [Ng+]	183 [Npl]
	7	863A	ASP	2.09	2.76	116.32	2,771 [Nam]	210 [O3]
	8	863A	ASP	2.56	3.23	123.39	233 [N3]	2,771 [Nam]
	9	863A	ASP	1.42	2.44	163.19	214 [N3]	2,778 [O-]
	10	863A	ASP	2.48	3.45	157.66	207 [N3]	2,778 [O-]
	11	866A	LEU	1.76	2.77	167.81	2,810 [Nam]	199 [O3]
	12	868A	ARG	2.49	2.99	109.67	2,849 [Ng+]	157 [O3]
	13	884A	VAL	3.2	4.05	142.25	2,945 [N3]	165 [N3]
	14	884A	VAL	3.37	4.05	126.23	165 [N3]	2,945 [N3]
	15	884A	VAL	2.46	2.92	106.99	132 [N3]	2,945 [N3]
Cont	1	801A	MET	2.26	3.22	163.32	695 [Nam]	2,218 [Nar]

As shown in Table 3, in hydrogen bonding, the distance between the donor and acceptor atoms typically ranges from 2.6 to 3.3 Å. According to Jeffrey’s classification, hydrogen bonds with donor-acceptor distances ranging from 2.2 to 2.5 Å are considered “strong, primarily covalent,” while those spanning 2.5–3.2 Å are labeled “moderate, predominantly electrostatic.” Bonds with distances of 3.2–4.0 Å are classified as “weak, primarily electrostatic” (Jeffrey and Jeffrey, 1997; Khan et al., 2022). The results are considered better than those obtained from the docking of the co-crystallized ligand, which generated only one hydrogen bond and two hydrophobic bonds.

### MD simulation analysis

**FIGURE 2:**
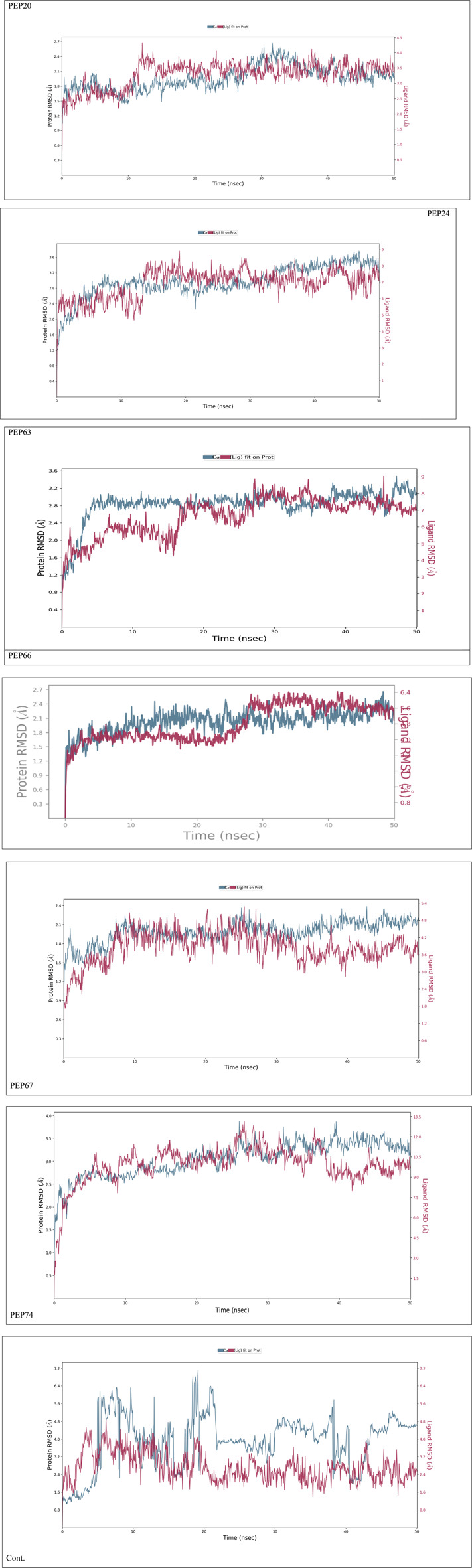
The RMSD analysis represents the root mean square deviation (RMSD) of peptides and control compound (in red) and HER2 protein backbones (in blue) over 50 nanoseconds of MD simulations. The RMSD values for both the peptide and the protein fluctuated over time, indicating their dynamic nature within the simulation environment.

**FIGURE 3:**
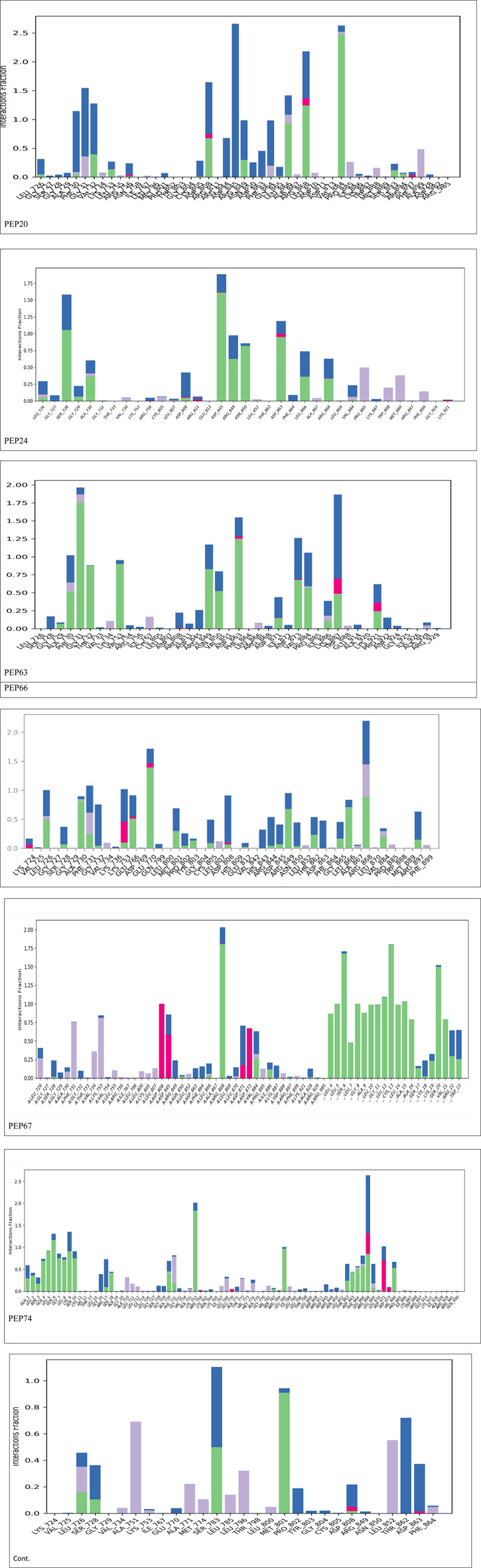
Histogram showing bonds generated from peptides and protein backbones. During the 50 ns MD simulations, green represents hydrogen bonds, blue represents water bridges, violet represents hydrophobic bonds, and red represents ionic bonds. The stacked bar charts are normalized throughout the trajectory: for instance, a value of 0.7 indicates that the specific interaction is maintained for 70% of the simulation time. Values exceeding 1.0 can occur because some protein residues may form multiple contacts of the same subtype with the ligand.

**FIGURE 4:**
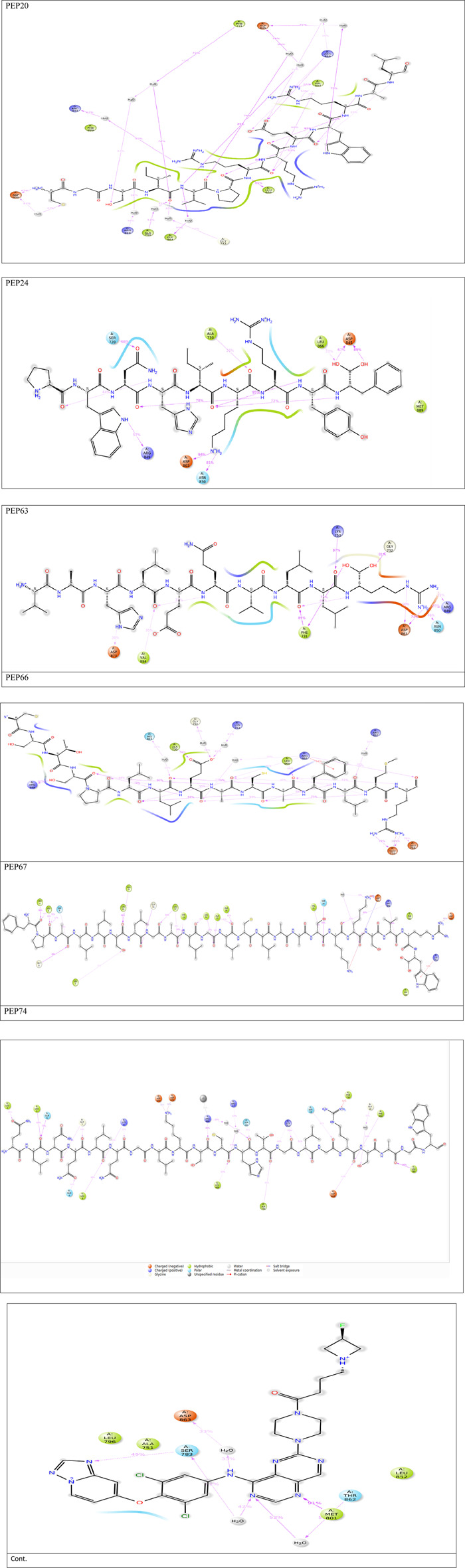
2D interactions generated from peptides and HER2 protein residues during 50 ns MD simulations.Only interactions that occur more than 30% of the simulation time (50 nsec) are displayed. Note that interactions can exceed 100% because some residues may form multiple interactions of the same type with a single ligand atom.

Figure 2 represents the root mean square deviation (RMSD) analysis of the peptides (in red) and HER2 protein backbones (in blue) over 50 nanoseconds of MD simulations. The RMSD values for both the peptide and the protein fluctuated over time, indicating their dynamic nature within the simulation environment. All peptides were aligned with the protein backbones from the beginning to the end of the simulation, and a slight deviation was observed for some peptides, but the deviation was still within the acceptable range (less than 3 Å) (Lee et al., 2000). These findings indicate the stability of the generated complexes. This alignment is supported by different types of bonds between amino acid residues and a protein backbone, as shown in Figure 3. Each color represents a type of bond: green for hydrogen bonds, blue for water bridges, violet for hydrophobic bonds, and red for ionic bonds. Residues with columns exceeding 0.7 are indicative of stable bonding (Charbe et al., 2024). Most of the peptides showed stable hydrogen bonds with the essential residues in the active site of HER2: SER728, PHE731, LYS753, ASP845, ARG849, ASN850, ARG860, ASP863, and LYS887 (Bello and Mejía, 2021; Wulandari et al., 2021). The higher stability of PEP66 aligned with the experimental results, in which the highest inhibition percentage was recorded. Several stable intermolecular interactions between PEP66 and HER2 at Lys753, Glu770, Arg849, and Arg868 were observed during the 50 ns simulation (Figures 3, 4). This finding may support the inhibitory role of PEP66, as it blocks the essential residue Lys753 located in the catalytic site, which coordinates with Mg2+ and ATP. Additionally, PEP66 may hinder the Arg849 residue, which is also located in the catalytic site and coordinates with Mg2+ and ATP (Collier et al., 2013). When compared to the control the peptides showed better stability during the whole period of simulation, as shown in Figure 2, the co-crystalized ligand separated from protein backbones and showed higher fluctuations compared to our peptides.

### Experimental procedure

**FIGURE 5:**
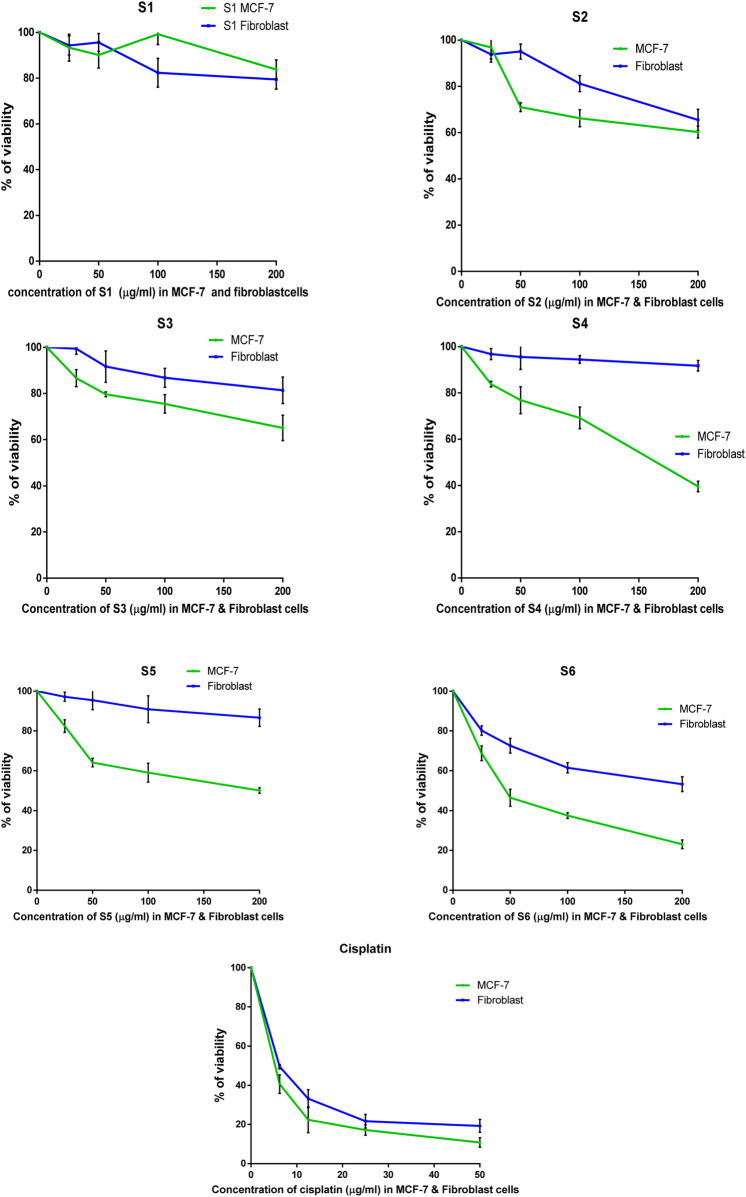
The percentage of surviving MCF-7 and HDFa after treatment with lactoferrin peptides (PEP67-S1, PEP74-S2, PEP24-S3, PEP63-S4, PEP20-S5, and PEP66-S6) obtained from camel milk.

The antitumor properties of lactoferrin peptides (PEP20, PEP24, PEP63, PEP66, PEP67 and PEP74) isolated from CM were assessed using the MTT assay on cancer MCF-7 cells and normal HDFa human line cells. The viability of these cells was determined in relation to various peptide concentrations (Figure 5). The IC_50_ values of the peptides were computed for both normal and breast cancer cells (Table 4).

**TABLE 4:** The IC_50s_ (μg/mL) of the peptides PEP67, PEP74, PEP24, PEP63, PEP20, and PEP66 against the cancer cell line MCF-7 and the normal cell line HDFa.

	PEP67	PEP74	PEP24	PEP63
MCF-7 Range Value SI	648.5–2092 1,165 ± 731 0.57	171.0–304.0 228 ± 66.7 1.86	241.7–383.9 304.6 ± 71.3 2.6	135.8–198.1 164 ± 31.2 11.7
HDFa Range value	498.0–899.9 669.4 ± 201	348.0–517.2 424.3 ± 84.7	580.4–1,073 789 ± 247	1,284 - 2,872 1920 ± 799

	PEP20	PEP66	Cisplatin	
MCF-7 Range Value SI	112.6–177.8 141.5 ± 32.6 8.38	46.73–59.71 52.82 ± 6.49 3.19	3.581–5.055 4.255 ± 0.738 1.565	
HDFa Range value	827.0–1701 1,186 ± 439	137.2–206.9 168.5 ± 34.9	5.650–7.856 6.663 ± 1.10	

The peptides PEP20, PEP24, PEP63, PEP66, PEP67, and PEP74 had corresponding IC50 values of 141.5, 304, 164, 1,165 and 228 μg/mL, respectively, in MCF7 cancer cells and 669, 424, 789, 1920, 1,186, and 168 μg/mL, respectively, in HDFa normal cells. Cisplatin demonstrated greater efficacy in MCF-7 cells and HDFAs. In MCF7 and HDFa cells, the IC50 values of cisplatin were 4.25 and 6.66 μg/mL, respectively. Compared to other peptides, PEP66 in MCF-7 cells had the lowest IC50 (52.82 μg/mL), as shown in Table 4, suggesting that it was the most effective against breast cancer. PEP63 and PEP20 had lower IC_50_ values (164, 141.5 μg/mL) than did PEP74 and PEP24 (228, 304 μg/mL). Compared to those of the other peptides in MCF-7 cells, the IC_50_ of PEP67 (1,165 μg/mL) was greater and attenuated in breast cancer cells. Although the IC_50_ of PEP66 in MCF-7 cells was the lowest, it was still 12 times greater than the IC_50_ of cisplatin (4.2 μg/mL).

While iron is required for normal cell division and growth, iron-free conditions increase DNA damage and oxidative stress, which leads to cancer. Lactoferrin binds iron, reducing the amount of free iron available and therefore the pro-carcinogenic effects of iron (Brown et al., 2020). The anti-inflammatory effects of lactoferrin help to stop the development and progression of cancer. Lactoferrin may aid the immune system in fighting against cancerous cells (Ohradanova-Repic et al., 2023). Treatment of cancer cells with lactoferrin disrupts the cytoskeleton, induces apoptosis, suppresses angiogenesis, reduces cell migration, and arrests the cell cycle. High concentrations of glycosaminoglycan, silicic acid, and proteoglycans in cells interact with lactoferrin, potentially upregulating E-cadherin and downregulating vimentin in oral squamous cell carcinoma (OSCC), reversing the epithelial-to-mesenchymal transition (EMT) process. On the surface of cells and the extracellular matrix, vimentin is detectable and influences cell migration. The tumor suppressor protein E-cadherin promotes cell-to-cell adhesion. Tumor cell E-cadherin expression is frequently lost during tumor growth and metastasis, frequently in conjunction with EMT (Chea et al., 2018; Cutone et al., 2020; Rascón-Cruz et al., 2021b).

**FIGURE 6:**
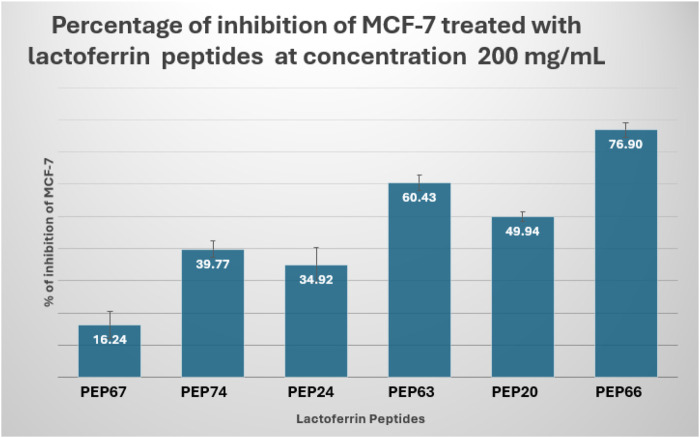
Percent inhibition of MCF-7 cells treated with lactoferrin peptides (PEP67, PEP74, PEP24, PEP63, PEP20 and PEP66) at a concentration of 200 mg/mL.

The results showed that PEP66 (77% ± 2.193) had the greatest inhibitory effect on MCF-7 cells after treatment with lactoferrin peptides at a dose of 200 μg/mL. The percentage of MCF7 inhibition was as follows: PEP63 (60% ± 2.2.83) > PEP20 (50% ± 1.389) > PEP74 (40% ± 2.569) > PEP24 (35% ± 5.499) (Figure 6).

The IC_50_ of peptides (PEP20, PEP24, PEP63, PEP66, PEP67 and PEP74) of normal HDFAs was allocated to the IC_50_ of these peptides in MCF-7 cells to calculate the selectivity index (SI) (Table 4). Significant toxicity is indicated by an SI value less than two (Swanepoel et al., 2019). With respect to toxicity, PEP63 (SI = 11.7) exhibited the lowest toxicity in MCF-7 cells. The second lowest toxicity was represented by the SI of PEP20 (SI = 8.38). Despite having the lowest IC_50_, PEP66 is nevertheless not significantly hazardous to normal HDFa, as indicated by its 3.16 SI index. PEP24 (2.6) is less hazardous than PEP74 (1.86). Cisplatin is toxic to normal HDFa cells, with an SI of 1.565 when applied to MCF-7 cells. PEP67 had the lowest value (0.6) and was more selective for normal HDFa cells than for breast MCF-7 cancer cells.

The proliferation of four breast cancer cell lines (T-47D, MDA-MB-231, Hs578T, and MCF-7) but not of the normal breast cell line MCF-10-2A is inhibited by bovine lactoferrin, indicating lactoferrin selectivity (Zhang et al., 2014). The lactoferrin selective indices against MDAmb231 and HeLa cells were 11.68 and 9.59, respectively, indicating that lactoferrin has high cytotoxic selectivity for cancer cells and a low harmful effect on healthy cells (Rascón-Cruz et al., 2021b).

## Conclusion

In conclusion, PEP66, PEP20, PEP63, PEP74, and PEP24 may have anticancer effects on breast cancer cell lines (MCF-7). PEP66 had the lowest IC50 value in MCF-7 cells, and the SI was 3.2, indicating that normal HDFa cells are less hazardous when given the same dose as cancer cells. The highest SI was observed for PEP63. As a potential anticancer agent, lactoferrin may lessen the negative effects of chemotherapy medications. For a full understanding of the processes by which lactoferrin induces apoptosis and may also undergo autophagy in cancer cells, additional *in vitro* and *in vivo* investigations are suggested.

### Limitations of the study

The detailed mechanisms by which these peptides induce anticancer effects are not fully elucidated. Further studies are required to understand the pathways involved. The long-term effects and potential toxicity of the peptides were not studied. Long-term studies are needed to evaluate the safety and efficacy of the peptides over extended periods. The study does not account for environmental factors that may influence peptide activity, such as pH levels, temperature variations, and the presence of other biomolecules in a real biological system. The study investigates a limited number of lactoferrin peptides. There may be other peptides within camel milk with significant anticancer properties that were not explored. Additional *in vivo* studies are required to better understand the complex environment of a living organism.

## Data Availability

The original contributions presented in the study are included in the article/supplementary material, further inquiries can be directed to the corresponding authors.

## Abbreviations

CM, Camel Milk; HER2, Human Epidermal Growth Factor Receptor 2; HDFa, Human Dermal Fibroblast Adult; IC50, Half Maximal Inhibitory Concentration; LF, Lactoferrin; MD, Molecular Dynamics; MTT, 3-(4,5-Dimethylthiazol-2-yl)-2,5-Diphenyltetrazolium Bromide; SVM, Support Vector Machine; ER, Estrogen Receptor; PR, Progesterone Receptor; EMT, Epithelial-to-Mesenchymal Transition; OSCC, Oral Squamous Cell Carcinoma; SI, Selectivity Index; TIP4P, Transferable Intermolecular Potential with 4 Points; OPLS3e, Optimized Potentials for Liquid Simulations 3 Enhanced; NPT, Constant Number of Particles, Pressure, and Temperature; PDB, Protein Data Bank; FBS, Fetal Bovine Serum; DMEM, Dulbecco’s Modified Eagle Medium; ATP, Adenosine Triphosphate; AA, Amino Acid; Mol wt, Molecular Weight; RMSD, Root Mean Square Deviation.

## References

[B1] Adasme M. F. Linnemann K. L. Bolz S. N. Kaiser F. Salentin S. Haupt V. J. (2021). PLIP 2021: expanding the scope of the protein–ligand interaction profiler to DNA and RNA. Nucleic acids Res. 49, W530–W534. 10.1093/nar/gkab294 33950214 PMC8262720

[B2] Agrawal P. Bhagat D. Mahalwal M. Sharma N. Raghava G. P. (2021). AntiCP 2.0: an updated model for predicting anticancer peptides. Briefings Bioinforma. 22, bbaa153. 10.1093/bib/bbaa153 32770192

[B3] Alguridi H. I. Alzahrani F. Almalki S. Zamzami M. A. Altayb H. N. (2023). Identification and molecular docking of novel chikungunya virus NSP4 inhibitory peptides from camel milk proteins. J. Biomol. Struct. Dyn., 1–16. 10.1080/07391102.2023.2254398 37668009

[B4] Ali E. M. Elashkar A. A. El-Kassas H. Y. Salim E. I. (2018). Methotrexate loaded on magnetite iron nanoparticles coated with chitosan: biosynthesis, characterization, and impact on human breast cancer MCF-7 cell line. Int. J. Biol. Macromol. 120, 1170–1180. 10.1016/j.ijbiomac.2018.08.118 30172815

[B5] Alkhulaifi M. M. Alosaimi M. M. Khan M. S. Tabrez S. Shaik G. M. Alokail M. S. (2024). Assessment of broad-spectrum antimicrobial, antibiofilm, and anticancer potential of lactoferrin extracted from camel milk. Appl. Biochem. Biotechnol. 196, 1464–1480. 10.1007/s12010-023-04579-7 37418128

[B6] Al Musaimi O. Lombardi L. Williams D. R. Albericio F. (2022). Strategies for improving peptide stability and delivery, Pharm. (Basel) Strategies Improv. Peptide Stab. Deliv. 15, 1283. 10.3390/ph15101283 PMC9610364 36297395

[B7] Asma S. T. Acaroz U. Imre K. Morar A. Shah S. R. A. Hussain S. Z. (2022). Natural products/bioactive compounds as a source of anticancer drugs. Cancers 14, 6203. 10.3390/cancers14246203 36551687 PMC9777303

[B8] Badani H. Garry R. F. Wimley W. C. (2014). Peptide entry inhibitors of enveloped viruses: the importance of interfacial hydrophobicity. Biochimica Biophysica Acta (BBA)-Biomembranes 1838, 2180–2197. 10.1016/j.bbamem.2014.04.015 PMC7094693 24780375

[B9] Baothman O. Ali E. M. M. Hosawi S. Hassan E. K. E. Abu Zeid I. M. Ahmad A. (2024). Prediction of anticancer peptides derived from the true lectins of Phoenix dactylifera and their synergetic effect with mitotane. Front. Pharmacol. 15, 1322865. 10.3389/fphar.2024.1322865 38464729 PMC10920327

[B10] Bello M. Mejía M. Á. V. (2021). “Structural insight of the anticancer properties of doxazosin on overexpressing EGFR/HER2 cell lines,” in Breast cancer-evolving challenges and next frontiers. IntechOpen.

[B11] Boopathi V. Subramaniyam S. Malik A. Lee G. Manavalan B. Yang D.-C. (2019). mACPpred: a support vector machine-based meta-predictor for identification of anticancer peptides. Int. J. Mol. Sci. 20, 1964. 10.3390/ijms20081964 31013619 PMC6514805

[B12] Bowers K. J. Chow E. Xu H. Dror R. O. Eastwood M. P. Gregersen B. A. (2006). “Scalable algorithms for molecular dynamics simulations on commodity clusters,” in Proceedings of the 2006 ACM/IEEE Conference on supercomputing, 84–es.

[B13] Bray F. Laversanne M. Sung H. Ferlay J. Siegel R. L. Soerjomataram I. (2024). Global cancer statistics 2022: GLOBOCAN estimates of incidence and mortality worldwide for 36 cancers in 185 countries. CA a cancer J. Clin. 74, 229–263. 10.3322/caac.21834 38572751

[B14] Brown R. A. Richardson K. L. Kabir T. D. Trinder D. Ganss R. Leedman P. J. (2020). Altered iron metabolism and impact in cancer biology, metastasis, and immunology. Front. Oncol. 10, 476. 10.3389/fonc.2020.00476 32328462 PMC7160331

[B15] Charbe N. B. Zacconi F. C. Kowthavarapu V. K. Gupta C. Palakurthi S. S. Satheeshkumar R. (2024). Targeting allosteric site of PCSK9 enzyme for the identification of small molecule inhibitors: an *in silico* drug repurposing study. Biomedicines 12, 286. 10.3390/biomedicines12020286 38397888 PMC10887305

[B16] Chea C. Miyauchi M. Inubushi T. Okamoto K. Haing S. Nguyen P. T. (2018). Bovine lactoferrin reverses programming of epithelial-to-mesenchymal transition to mesenchymal-to-epithelial transition in oral squamous cell carcinoma. Biochem. biophysical Res. Commun. 507, 142–147. 10.1016/j.bbrc.2018.10.193 30415774

[B17] Collier T. S. Diraviyam K. Monsey J. Shen W. Sept D. Bose R. (2013). Carboxyl group footprinting mass spectrometry and molecular dynamics identify key interactions in the HER2-HER3 receptor tyrosine kinase interface. J. Biol. Chem. 288 **,** 25254–25264. 10.1074/jbc.M113.474882 23843458 PMC3757188

[B18] Cutone A. Rosa L. Ianiro G. Lepanto M. S. Bonaccorsi Di Patti M. C. Valenti P. (2020). Lactoferrin’s anti-cancer properties: safety, selectivity, and wide range of action. Biomolecules 10, 456. 10.3390/biom10030456 32183434 PMC7175311

[B19] El-Bendary M. M. Akhdhar A. Al-Bogami A. S. Domyati D. Kalantan A. A. Alzahrani F. A. (2024). Palladium and platinum complexes based on pyridine bases induced anticancer effectiveness via apoptosis protein signaling in cancer cells. BioMetals 37, 905–921. 10.1007/s10534-023-00580-z 38361146

[B20] El-Fakharany E. M. Abu-Serie M. M. Habashy N. H. Eltarahony M. (2022). Augmenting apoptosis-mediated anticancer activity of lactoperoxidase and lactoferrin by nanocombination with copper and iron hybrid nanometals. Sci. Rep. 12, 13153. 10.1038/s41598-022-17357-y 35915221 PMC9343395

[B21] Fatemi M. Ghandehari F. Bahrami S. Tajedin N. (2018). *In silico* and *in vitro* studies of cytotoxic activity of different peptides derived from human lactoferrin protein. J. Kermanshah Univ. Med. Sci. 22. 10.5812/jkums.69544

[B22] Jeffrey G. A. Jeffrey G. A. (1997). An introduction to hydrogen bonding. New York: Oxford University Press.

[B23] Kaskous S. (2016). Importance of camel milk for human health. Emir. J. Food Agric. 28, 158. 10.9755/ejfa.2015-05-296

[B24] Khan I. M. Islam M. Shakya S. Alam N. Imtiaz S. Islam M. R. (2022). Synthesis, spectroscopic characterization, antimicrobial activity, molecular docking and DFT studies of proton transfer (H-bonded) complex of 8-aminoquinoline (donor) with chloranilic acid (acceptor). J. Biomol. Struct. Dyn. 40, 12194–12208. 10.1080/07391102.2021.1969280 34473009

[B25] Khan M. Z. Xiao J. Ma Y. Ma J. Liu S. Khan A. (2021). Research development on anti-microbial and antioxidant properties of camel milk and its role as an anti-cancer and anti-hepatitis agent. Antioxidants (Basel). 10, 788. 10.3390/antiox10050788 34067516 PMC8156492

[B26] Konozy E. H. E. Osman M. E. M. (2022). Plant lectin: a promising future anti-tumor drug. Biochimie 202, 136–145. 10.1016/j.biochi.2022.08.002 35952948

[B27] Krishnankutty R. Iskandarani A. Therachiyil L. Uddin S. Azizi F. Kulinski M. (2018). Anticancer activity of camel milk via induction of autophagic death in human colorectal and breast cancer cells. Asian Pac J. Cancer Prev. 19, 3501–3509. 10.31557/APJCP.2018.19.12.3501 30583676 PMC6428541

[B28] Kumar R. Mandal M. Lipton A. Harvey H. Thompson C. B. (1996). Overexpression of HER2 modulates bcl-2, bcl-XL, and tamoxifen-induced apoptosis in human MCF-7 breast cancer cells. Clin. cancer Res. official J. Am. Assoc. Cancer Res. 2, 1215–1219. 9816290

[B29] Lattrich C. Juhasz-Boess I. Ortmann O. Treeck O. (2008). Detection of an elevated HER2 expression in MCF-7 breast cancer cells overexpressing estrogen receptor beta1. Oncol. Rep. 19, 811–817. 18288420

[B30] Lee K.-H. Benson D. R. Kuczera K. (2000). Transitions from alpha to pi helix observed in molecular dynamics simulations of synthetic peptides. Biochemistry 39, 13737–13747. 10.1021/bi001126b 11076513

[B31] Liu X. Wu F. Ji Y. Yin L. (2019). Recent advances in anti-cancer protein/peptide delivery. Bioconjugate Chem. 30, 305–324. 10.1021/acs.bioconjchem.8b00750 30428665

[B32] Malik A. Al-Senaidy A. Skrzypczak-Jankun E. Jankun J. (2012). A study of the anti-diabetic agents of camel milk. Int. J. Mol. Med. 30, 585–592. 10.3892/ijmm.2012.1051 22751901

[B33] Meng E. C. Goddard T. D. Pettersen E. F. Couch G. S. Pearson Z. J. Morris J. H. (2023). UCSF ChimeraX: tools for structure building and analysis. Protein Sci. 32, e4792. 10.1002/pro.4792 37774136 PMC10588335

[B34] Ohradanova-Repic A. Praženicová R. Gebetsberger L. Moskalets T. Skrabana R. Cehlar O. (2023). Time to kill and time to heal: the multifaceted role of lactoferrin and lactoferricin in host defense. Pharmaceutics 15, 1056. 10.3390/pharmaceutics15041056 37111542 PMC10146187

[B35] Rascón-Cruz Q. Espinoza-Sánchez E. A. Siqueiros-Cendón T. S. Nakamura-Bencomo S. I. Arévalo-Gallegos S. Iglesias-Figueroa B. F. (2021a). Lactoferrin: a glycoprotein involved in immunomodulation, anticancer, and antimicrobial processes, Mol. Lactoferrin A Glycoprotein Involv. Immunomodulation, Anticancer, Antimicrob. Process. 26, 205. 10.3390/molecules26010205 PMC7795860 33401580

[B36] Rascón-Cruz Q. Espinoza-Sánchez E. A. Siqueiros-Cendón T. S. Nakamura-Bencomo S. I. Arévalo-Gallegos S. Iglesias-Figueroa B. F. (2021b). Lactoferrin: a glycoprotein involved in immunomodulation, anticancer, and antimicrobial processes. Molecules 26, 205. 10.3390/molecules26010205 33401580 PMC7795860

[B37] Saikia S. Bordoloi M. (2019). Molecular docking: challenges, advances and its use in drug discovery perspective. Curr. drug targets 20, 501–521. 10.2174/1389450119666181022153016 30360733

[B38] Salim E. I. Abd El Khalik E. A. Shalaby T. I. Ali E. M. (2022). Synthesis, characterisation and enhanced apoptotic effect of gemcitabine-loaded albumin nanoparticles coating with chitosan. Archives Physiology Biochem. 128, 970–978. 10.1080/13813455.2020.1742165 32212969

[B39] Sitaram N. Nagaraj R. (1999). Interaction of antimicrobial peptides with biological and model membranes: structural and charge requirements for activity. Biochimica Biophysica Acta (BBA)-Biomembranes 1462, 29–54. 10.1016/s0005-2736(99)00199-6 10590301

[B40] Superti F. (2020). Lactoferrin from bovine milk: a protective companion for life. Nutrients 12, 2562. 10.3390/nu12092562 32847014 PMC7551115

[B41] Swelum A. A. El-Saadony M. T. Abdo M. Ombarak R. A. Hussein E. O. S. Suliman G. (2021). Nutritional, antimicrobial and medicinal properties of Camel's milk: a review. Saudi J. Biol. Sci. 28, 3126–3136. 10.1016/j.sjbs.2021.02.057 34025186 PMC8117040

[B42] Taghipour M. J. Ezzatpanah H. Ghahderijani M. (2023). *In vitro* and *in silico* studies for the identification of anti-cancer and antibacterial peptides from camel milk protein hydrolysates. Plos one 18, e0288260. 10.1371/journal.pone.0288260 37437001 PMC10337890

[B43] Tan K. P. Singh K. Hazra A. Madhusudhan M. S. (2021). Peptide bond planarity constrains hydrogen bond geometry and influences secondary structure conformations. Curr. Res. Struct. Biol. 3, 1–8. 10.1016/j.crstbi.2020.11.002 34382009 PMC8261469

[B44] Teerasak E. Thongararm P. Roytrakul S. Meesuk L. Chumnanpuen P. (2016). Prediction of anticancer peptides against MCF-7 breast cancer cells from the peptidomes of *Achatina fulica* mucus fractions. Comput. Struct. Biotechnol. J. 14, 49–57. 10.1016/j.csbj.2015.11.005 26862373 PMC4706611

[B45] Wadood A. Ahmed N. Shah L. Ahmad A. Hassan H. Shams S. (2013). In-silico drug design: an approach which revolutionarised the drug discovery process. OA Drug Des. Deliv. 1, 3. 10.13172/2054-4057-1-1-1119

[B46] Wilding B. Scharn D. Böse D. Baum A. Santoro V. Chetta P. (2022). Discovery of potent and selective HER2 inhibitors with efficacy against HER2 exon 20 insertion-driven tumors, which preserve wild-type EGFR signaling. Nat. Cancer 3, 821–836. 10.1038/s43018-022-00412-y 35883003

[B47] Wulandari A. A. Choiri A. A. Widiandani T. (2021). Thymoquinone and its derivatives against breast cancer with HER2 positive: *in silico* studies of ADMET, docking and QSPR. J. Basic Clin. Physiology Pharmacol. 32, 393–401. 10.1515/jbcpp-2020-0431 34214298

[B48] Zhang Y. Nicolau A. Lima C. F. Rodrigues L. R. (2014). Bovine lactoferrin induces cell cycle arrest and inhibits mTOR signaling in breast cancer cells. Nutr. cancer 66, 1371–1385. 10.1080/01635581.2014.956260 25356800

[B49] Zhou P. Jin B. Li H. Huang S.-Y. (2018). HPEPDOCK: a web server for blind peptide–protein docking based on a hierarchical algorithm. Nucleic acids Res. 46, W443–W450. 10.1093/nar/gky357 29746661 PMC6030929

